# Prognostic analysis of patients with non-small cell lung cancer harboring exon 19 or 21 mutation in the epidermal growth factor gene and brain metastases

**DOI:** 10.1186/s12885-020-07249-7

**Published:** 2020-09-03

**Authors:** Jing Wang, Zhiyan Liu, Qingsong Pang, Tian Zhang, Xi Chen, Puchun Er, Yuwen Wang, Ping Wang, Jun Wang

**Affiliations:** Department of Radiation Oncology, Tianjin Medical University Cancer Institute and Hospital, Key Laboratory of Cancer Prevention and Therapy, National Clinical Research Center for Cance, Tianjin’s Clinical Research Centre for Cancer, Huan-Hu-Xi Road, Ti-Yuan-Bei, He Xi District, Tianjin, 300060 PR China

**Keywords:** Non-small cell lung cancer, Brain metastasis, *epidermal growth factor receptor* mutation, Prognosis, Treatment

## Abstract

**Background:**

In 1997, the Radiation Therapy Oncology Group (RTOG) put forward the recursive partitioning analysis classification for the prognosis of brain metastases (BMs), but this system does not take into account the *epidermal growth factor receptor* (*EGFR*) mutations. The aim of the study is to assess the prognosis of patients with *EGFR*-mutated non-small cell lung cancer (NSCLC) and BMs in the era of tyrosine kinase inhibitor (TKI) availability.

**Methods:**

This was a retrospective study of consecutive patients with *EGFR*-mutated (exon 19 or 21) NSCLC diagnosed between 01/2011 and 12/2014 at the Tianjin Medical University Cancer Institute & Hospital and who were ultimately diagnosed with BMs. The patients were stage I-III at initial presentation and developed BMs as the first progression. Overall survival (OS), OS after BM diagnosis (mOS), intracranial progression-free survival (iPFS), response to treatment, and adverse reactions were analyzed.

**Results:**

Median survival was 35 months, and the 1- and 2- year survival rates were 95.6% (108/113) and 74.3% (84/113). The 3-month CR + PR rates of radiotherapy(R), chemotherapy(C), targeted treatment(T), and targeted treatment + radiotherapy(T+R) after BMs were 63.0% (17/27), 26.7% (4/15), 50.0% (7/14), and 89.7% (35/39), respectively. The median survival of the four treatments was 20, 9, 12, and 25 months after BMs, respectively (*P* = 0.001). Multivariable analysis showed that < 3 BMs (odds ratio (OR) = 3.34, 95% confidence interval (CI): 1.89–5.91, *P* < 0.001) and treatment after BMs (OR = 0.68, 95%CI: 0.54–0.85, *P* = 0.001) were independently associated with better prognosis.

**Conclusions:**

The prognosis of patients with NSCLC and EGFR mutation in exon 19 or 21 after BM is associated with the number of brain metastasis and the treatment method. Targeted treatment combined with radiotherapy may have some advantages over other treatments, but further study is warranted to validate the results.

## Background

Lung cancer is the cancer with the world’s highest morbidity and mortality [1]. Non-small cell lung cancer (NSCLC) accounts for 80–85% of the cases of lung cancer. NSCLC mainly affects adults > 65 years old, tobacco smokers, and men [2, 3]. In China, in 2014, approximately 2,114,000 men and 1,690,000 women have been diagnosed with lung cancer, representing 10,422 new cases each day; in addition, there were 2,296,000 deaths attributable to lung cancer in 2014 [4].

Mutation in the *epidermal growth factor receptor* (*EGFR*) gene is now a key target in the treatment of NSCLC. Indeed, afatinib, erlotinib, gefitinib, icotinib, and osimertinib have been shown to improve the prognosis and survival of patients harboring *EGFR* sensitizing mutations [2, 5].

Brain metastases (BMs) are the main form of distant metastases in lung cancer and is one of the main causes of treatment failure [2, 3]. Approximately 25% of patients with NSCLC suffer from BM, and its occurrence influences survival [2, 3]. As early as 1997, the Radiation Therapy Oncology Group (RTOG) put forward a recursive partitioning analysis (RPA) for the classification of BMs [6], which was the first prognostic scoring system for assessing the prognosis of patients with BM, but this system does not take into account the presence of *EGFR* mutations. The therapeutic modalities to control BMs include whole-brain radiotherapy (WBRT), stereotactic radiosurgery (SRS), surgery, and chemotherapy, and the best approach has to be tailored to each patient based on the number of lesions, their size, their exact location, and the extent of invasion [2, 3]. Furthermore, the optimal treatment is unknown for *EGFR*-mutated patients with NSCLC and BMs [7], and this important research question remains to be examined.

Therefore, the aim of the present study was to assess the prognosis of patients with *EGFR*-mutated NSCLC and BMs in the era of TKI availability (except osimertinib, which was not available during the study period).

## Methods

### Study design and patients

This was a retrospective study of the consecutive patients with stage I-III NSCLC diagnosed between January 2011 and December 2014 at the Tianjin Medical University Cancer Institute & Hospital and who were ultimately diagnosed with BMs. The study was carried out in accordance with the Declaration of Helsinki and was approved by the ethics committee of Tianjin Medical University Cancer Institute & Hospital. The need for individual consent was waived by the committee.

The inclusion criteria were: 1) stage I-III NSCLC at initial diagnosis; 2) eligible to surgery and underwent radical surgery; 3) diagnosis confirmed by postoperative pathological examination; 4) confirmed with exon 19 deletion and exon 21 L858R missense mutation of EGFR using the surgical specimen after radical surgery; 5) did not have BMs before or after radical surgery; and 6) developed BMs during routine follow-up as the first progression. The patients with meningeal metastases were excluded.

### Treatments

All patients accepted standard lung cancer radical surgeries and adjuvant treatment according to the current guidelines at the time of their initial diagnosis. The diagnosis of BM was made based on enhanced head magnetic resonance (MRI) results. All patients had at least one measurable lesion (excluding patients with meningeal metastases).

The treatment options for BMs were: chemotherapy, radiotherapy, targeted therapy, and targeted therapy combined with radiotherapy. For radiotherapy, WBRT (40 Gy in 20 fractions or 30 Gy in 10 fractions) and/or SRS were conducted. For targeted therapy, gefitinib (250 mg, oral, once/day), erlotinib (150 mg, oral, once/day), or icotinib (125 mg, oral, three times/day) was used. The treatments were conducted until disease progression, death, or intolerable adverse reactions. The treatment selection was performed by a discussion between the patient and the physician. All cases were discussed at tumor boards. Some patients refused treatments because of costs since TKIs were expensive and not reimbursed by all insurance providers in China during the study period.

### Evaluation criteria

Overall survival (OS) was defined as the time from disease diagnosis to death or last follow-up. Overall survival after BM diagnosis (mOS) was the time from the diagnosis of BM to death or last follow-up. We defined intracranial progression-free survival (iPFS) as the interval between the diagnosis of BM and intracranial progression or mortality from any cause [8, 9]. The therapeutic effects were evaluated at 3 months using the RECIST criteria [10]. The therapeutic effect was classified as complete response (CR), partial response (PR), stable disease (SD), and progressive disease (PD). The objective response rate (ORR) was CR + PR. Toxicity was routinely documented according to the Common Terminology Criteria for Adverse Events (CTCAE) 3.0 [11].

### Data collection

All data were collected from the medical charts. The baseline characteristics were those at the time of BM diagnosis. The symptoms of BMs included dizziness, headache, nausea, vomiting, restricted limb activities, and unsteady walking.

### Statistical analysis

The continuous data were tested with the Kolmogorov-Smirnov test for normal distribution. Normally distributed continuous data are described as means ± standard deviation and were analyzed using the Student t-test or ANOVA with Tukey’s post hoc test, as appropriate. Skewed continuous variables are presented as median (range) and were analyzed using the Mann-Whitney U test or the Kruskal-Wallis test, as appropriate. The categorical variables are presented as frequencies and percentages and were analyzed using the chi-square test. The curves for OS, iPFS, and mOS were plotted using the Kaplan-Meier method, and comparisons between groups were calculated using the log-rank test. Multivariable analysis was carried out using Cox proportional hazard models (enter method) using variables that were significant in univariable analyses. *P* values < 0.05 were considered statistically significant. SPSS 18.0 for Windows (IBM, Armonk, NY, USA) was used for statistical analysis.

## Results

### Patient characteristics

From 560 patients with NSCLC who underwent radical resection and *EGFR* mutation testing, 113 (20.2%) with exon 19 deletion and exon 21 L858R missense mutation of EGFR and developed BMs as the first progression were included in this study. All cases were adenocarcinomas. Their median follow-up time was 30 months. Of the included cases, 44/113 cases were male (38.9%), and 69/113 cases were female (61.1%). The median age at onset was 58 (range, 31–79) years, with 91/113 (80.5%) patients being 65 years of age or younger, and 42/113 (37.2%) were smokers. Thirty patients received WBRT, 63 patients received stereotactic ablative radiotherapy (SABR), and 20 patients received a combination of WBRT and SABR. Regarding mutations, there were 52/113 (46.0%) cases of mutation in exon 19 and 61/113 (54.0%) of mutation in exon 21. The numbers of patients with stage I, II, and III NSCLC were 50/113 (44.2%), 11/113 (9.7%), and 52/113 (46.0%), respectively.

After being confirmed with BMs, 95/113 (84.1%) patients received further treatments: chemotherapy for 15/95 patients (15.8%), radiotherapy for 27/95 (28.4%), targeted therapy for 14/95 (14.7%), and targeted therapy combined with radiotherapy for 39/95 (41.1%).

### Treatment response

The proportion of patients with a complete or partial response after BM was significantly different across the treatment groups (*P* < 0.05) (Table 1). The proportion of CR + PR was 63.0% (17/27) for radiotherapy, 26.7% (4/15) for chemotherapy, 50.0% (7/14) for targeted therapy, and 89.7% (35/39) for targeted therapy combined with radiotherapy. Among those who received targeted therapy, gefitinib was used in 20 patients, erlotinib was used in 25 patients, and icotinib was used in 8 patients.

**Table 1:** Relation between short-term response across different treatments after BMs (*n* = 113)

		Response					P
		Complete	Partial	Stable	Progressive	Objective response rate	
Treatment	n	Response	Response	Disease	Disease		
None	18	0	0	9 (50.0%)	9 (50.0%)	0	< 0.05
Chemotherapy	15	0	4 (26.7%)	6 (40.0%)	5 (33.3%)	4 (26.7%)	
Radiotherapy	27	5 (18.5%)	12 (44.4%)	6 (22.2%)	4 (14.8%)	17 (63.0%)	
Targeted	14	1 (7.1%)	6 (42.9%)	5 (35.7%)	2 (14.3%)	7 (50.0%)	
Targeted combined radiotherapy	39	13 (33.3%)	22 (56.4%)	3 (7.7%)	1 (2.6%)	35 (89.7%)	

### Follow-up and survival

**Fig. 1:**
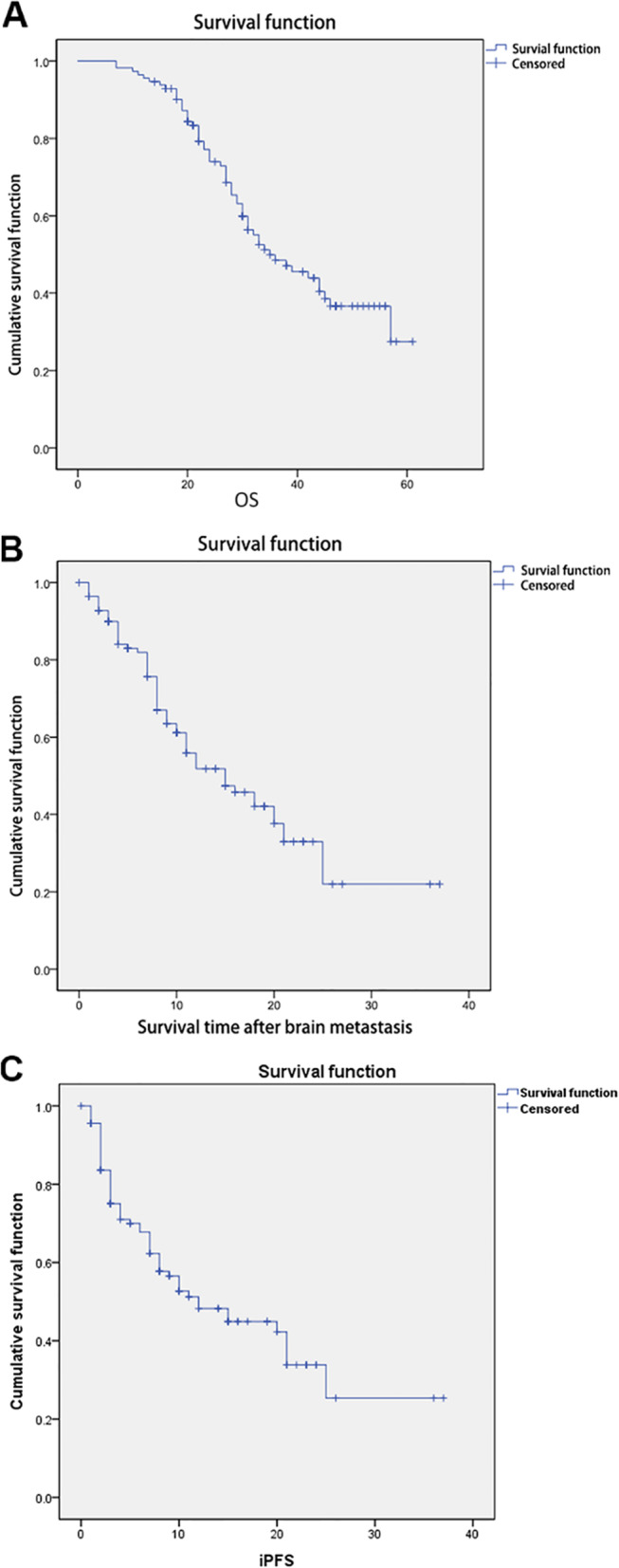
Survival analysis of patients with non-small cell lung cancer (NSCLC) and brain metastases (BMs). **a** Overall survival (OS). **b** Overall survival after BM diagnosis (mOS). **c** intracranial progression-free survival (iPFS)

All patients only had BMs when they entered this study. Subsequently, among all patients, as of the end of follow-up or death, a total of 61 patients had extracranial metastasis (including 36 bone metastases, 10 liver metastases, eight lung metastases, and two adrenal metastases) or malignant pleural effusions (*n* = 5). In 15 patients, local recurrence occurred (including primary foci and regional lymph nodes). The median OS was 35 months (range, 25.8–44.2 months), the one-year survival rate was 95.6%, and the two-year survival rate was 74.0% (Fig. 1A). The median time to BM was 17 months (range, 9.6–20.4 months) after the initial diagnosis of NSCLC. The median mOS was 15 months, and the one-year survival rate was 51.8% (Fig. 1B). The median iPFS was 12 months (range, 7.2–16.8 months), and the rate of intracranial progression in 1 year was 48.3% (Fig. 1C).

### Univariable analyses

Univariable analyses were performed to determine whether there were associations between clinical factors and mOS (Table 2). The results indicated that the ECOG score after the diagnosis of BM, the number of BMs, and the treatment after BM were associated with mOS.

**Table 2:** Univariable analyses of overall survival after BM among patients with EGFR-mutated NSCLC (*n* = 113)

Factors	n (%)	P	95% CI
Sex			
Male	44 (38.9%)	0.38	0.47–1.34
Female	69 (61.1%)		
Age			
≤ 65 years	91 (80.5%)	0.52	0.67–2.24
> 65 years	22 (19.5%)		
Histological type			
Adenocarcinoma	113 (100%)	–	–
*Epidermal growth factor receptor* gene mutation			
Exon 19	52 (46.0%)	0.13	0.94–1.61
Exon 21	61 (54.0%)		
Number of brain metastases			
≤ 3	57 (50.3%)	< 0.01	1.72–5.30
> 3	56 (49.6%)		
Maximum size of brain metastases			
≤ 2 cm	81 (71.7%)	0.33	0.75–2.36
> 2 cm	32 (28.3%)		
Symptoms associated with brain metastasis			
No	67 (59.3%)	0.62	0.51–1.50
Yes	46 (40.7)		
ECOG score			
≤ 2	83 (73.5%)	< 0.01	2.84–8.11
> 2	30 (26.5%)		
Treatment			
None	18 (15.9%)	< 0.01	0.55–0.86
Radiotherapy	27 (23.9%)		
Targeted therapy in previously TKI-naïve patients	14 (12.4%)		
Chemotherapy	15 (13.3%)		
Targeted combined radiotherapy	39 (34.5%)		

Abbreviation: *ECOG* Eastern Cooperative Oncology Group

### Multivariable analysis

**Fig. 2:**
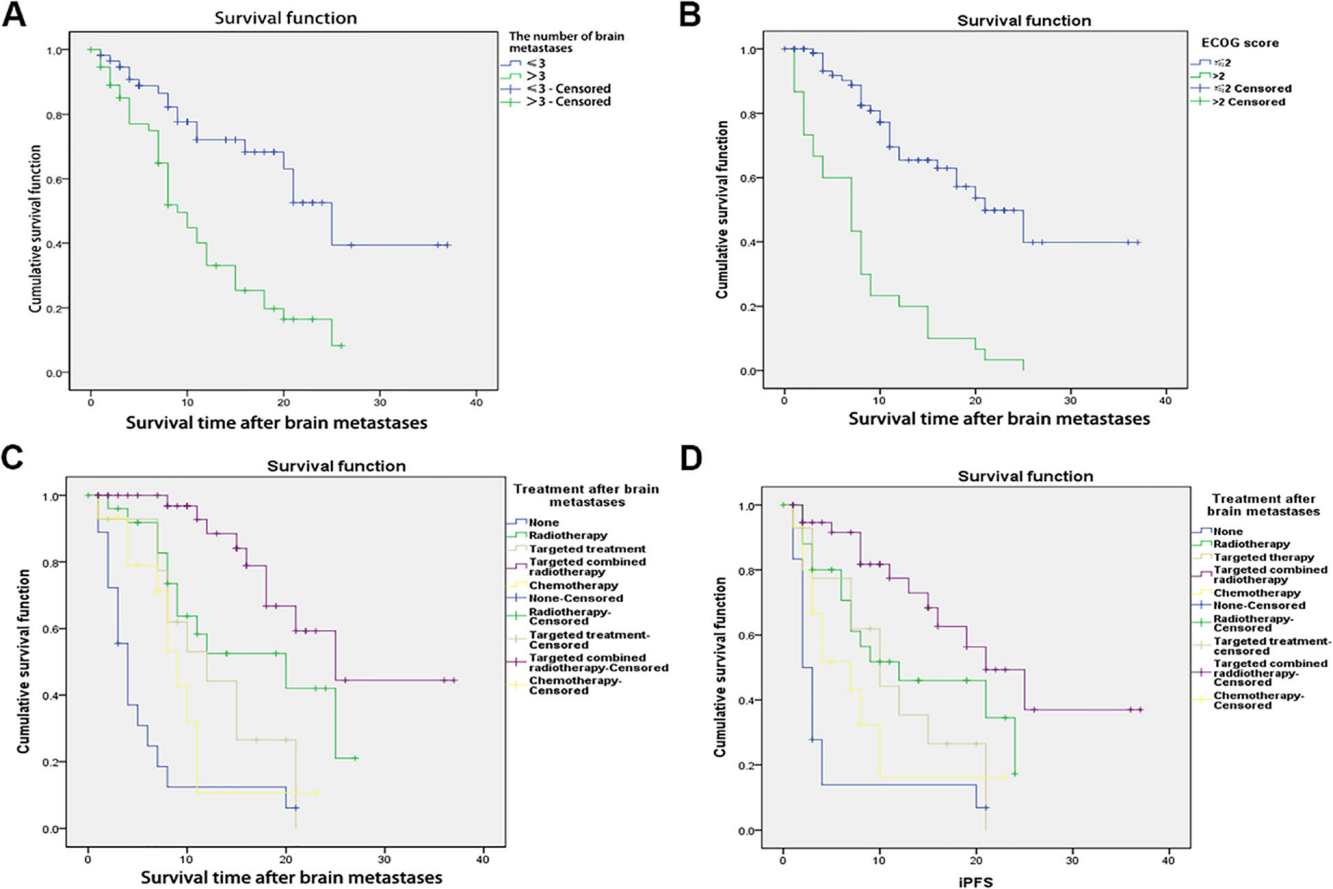
Survival of patients according to clinical characteristics. **a** Patients with < 3 brain metastases (BMs) showed survival advantage compared with those with > 3 BMs (25 (193.4–30.6) vs. 9 (6.9–11.1) months, *P* < 0.001). **b** Patients with ECOG score ≤ 2 showed a survival advantage compared with those with ECOG > 2 (21 (14.8–27.2) vs. 7 (3.8–10.2), *P <* 0.001). **c** After BMs, the median survival of the four groups of treatment was 20 (range, 6.0–34.0) months for radiotherapy, 9 (range, 7.0–11.1) months for chemotherapy, 12 (range, 5.7–18.3) months for targeted therapy, and 25 (range, 16.7–33.3) months for targeted therapy combined with radiotherapy (*P* < 0.05). **d** The median intracranial progression-free survival (iPFS) among the four treatments was 12 (range, 0–24.6) months for radiotherapy, 7 (range, 2.5–11.5) months for chemotherapy, 10 (range, 5.3–14.7) months for targeted therapy, and 21 (range, 14.0–28.0) months for targeted therapy combined with radiotherapy (*P* < 0.05)

Cox regression analysis was used to examine the association between risk factors identified in the univariable analyses with mOS (Table 3). Three or less intracranial metastases (*P* < 0.001) (Fig. 2a) and treatment after BM diagnosis (*P* = 0.001) (Fig. 2c-d) were independently associated with better mOS, while ECOG (Fig. 2b) was not.

**Table 3:** Multivariable analysis of the association between clinical factors and mOS in patients with NSCLC with *EGFR* mutation and BMs (*n =* 113)

Parameters	P	Odds ratio	95.0% CI for Exp(B)	
			Lower	Upper
ECOG score	0.080	1.481	0.953	2.301
Number of brain metastases	< 0.001	3.341	1.890	5.905
Treatments after brain metastases	0.001	0.680	0.543	0.851

Abbreviations: *CI* confidence interval, *ECOG* Eastern Cooperative Oncology Group

### Adverse reactions

Of all the patients, no grade 4–5 adverse reactions occurred. Of the group of patients with targeted therapy combined radiotherapy, no intolerable side effects leading to treatment discontinuation occurred. For chemotherapy, the most common adverse reaction was weakness. For radiotherapy, the most common adverse reaction was also weakness. For targeted therapy, the most common adverse reaction was rash. For targeted therapy combined with radiotherapy, the most common adverse reaction was weakness (Table 4).

**Table 4:** Toxicity grading of different treatments after BMs, n (%)

	Chemotherapy			Radiotherapy			Targeted therapy			Targeted combined radiotherapy		
	Grade 1	Grade 2	Grade 3	Grade 1	Grade 2	Grade 3	Grade 1	Grade 2	Grade 3	Grade 1	Grade 2	Grade 3
Weakness	10 66.7	5 33.3	0 0.0	12 44.4	4 14.8	0 0.0	0 0.0	0 0.0	0 0.0	20 51.3	5 12.8	0 0.0
Weight loss	8 53.3	2 13.3	0 0.0	5 18.5	0 0.0	0 0.0	0 0.0	0 0.0	0 0.0	8 20.5	0 0.0	0 0.0
Rash	0 0.0	0 0.0	0 0.0	0 0.0	0 0.0	0 0.0	7 50.0	3 21.4	0 0.0	16 41.0	3 7.7	0 0.0
Nausea	8 53.3	2 13.3	0 0.0	10 27.0	0 0.0	0 0.0	4 28.6	0 0.0	0 0.0	10 25.6	1 2.6	0 0.0
Vomiting	5 33.3	2 13.3	0 0.0	1 3.7	0 0.0	0 0.0	0 0.0	0 0.0v	0 0.0	2 5.1	0 0.0	0 0.0
Diarrhea	4 26.7	0 0.0	0 0.0	0 0.0	0 0.0	0 0.0	4 28.6	0 0.0	0 0.0	8 20.5	0 0.0	0 0.0v
Decreased absolute neutrophils value	5 33.3	1 6.7	1 6.7	5 18.5	1 3.7	0 0.0	0 0.0	0 0.0	0 0.0	6 15.4	2 5.1	0 0.0
Elevated ALT/AST	1 6.7	1 6.7	0 0.0	0 0.0	0 0.0	0 0.0	3 21.4	1 7.1	0 0.0	7 17.9	2 5.1	0 0.0
Elevated bilirubin	1 6.7	0 0.0	0 0.0	0 0.0	0 0.0	0 0.0	5 35.7	0 0.0	0 0.0	4 10.3	0 0.0	0 0.0
Headache	0 0.0	0 0.0	0 0.0	9 33.3	1 3.7	0 0.0	0 0.0	0 0.0	0 0.0	12 30.8	4 10.3	0 0.0
Dizziness	0 0.0	0 0.0	0 0.0	8 29.6	0 0.0	0 0.0	0 0.0	0 0.0	0 0.0	10 25.6	0 0.0	0 0.0

## Discussion

Many patients with lung cancer develop BMs, which impacts the quality of life and shortens survival. Despite therapy, the prognosis of NSCLC patients with BMs is poor, and the 1-year survival rate is < 20% [12]. Previous studies found a significant association between EGFR mutations and the risk of BM [13, 14] and pointed out the distinct clinical features of EGFR-mutated tumors in terms of BM [15–18]. Therefore, it is speculated that BMs in these patients exhibit their own characteristics in occurrence, treatment, and prognosis. In 1997, the RTOG put forward the recursive partitioning analysis classification for the prognosis of BMs, but this system does not take into account the *epidermal growth factor receptor* (*EGFR*) mutations present. Accordingly, this study aimed to summarize the factors affecting the prognosis of these patients with EGFR-mutated lung adenocarcinoma after BM. Furthermore, this study explored the optimal treatment for these patients.

Our results indicated that the number of BMs and treatment after BM were associated with overall survival after BMs. Previous studies concluded that the performance status [6, 19–21], age [6, 19–21], extracranial metastases [6, 19–21], and primary tumor control [19, 20] affected survival. Other studies [12, 22, 23] indicated that the number of BMs influenced survival. The choice of treatment should be based on the current guidelines and tailored to the clinical reality of each patient. Better physical strength generally means better tolerance. Nevertheless, our results were different from previous studies, probably because previous studies did not target patients with NSCLC harboring *EGFR* mutation and BMs. Few studies discussed the treatment factors influencing the prognosis of patients with BM and *EGFR* mutation. Gong et al [24]. indicated that the number of chemotherapy cycles and combined targeted therapy was key prognostic factors influencing survival. Our results indicate that the treatments after BM were associated with mOS. Due to the relatively small number of patients in each group, we were unable to exhaustively assess the factors that were correlated with the prognosis of patients with BM.

The first-generation EGFR-TKIs available in China during the study period included gefitinib, erlotinib, and icotinib. The CTONG0901 study compared the PFS and OS of gefitinib and erlotinib and found that the two were equivalent [25]. The ICOGEN study was a randomized, controlled phase III clinical trial comparing gefitinib to icotinib in previously treated patients with locally advanced or metastatic non-small cell lung cancer. The results showed that there was no significant difference in PFS and OS between gefitinib and icotinib [26]. The WJOG5108L clinical trial also showed that gefitinib and erlotinib were equivalent in PFS [27]. In clinical practice, which TKI a patient chooses is related to the patient’s choice and the doctor’s prescription habits. In the present study, gefitinib was used in 20 patients, erlotinib was used in 25 patients, and icotinib was used in 8 patients. Due to the price advantage of icotinib, some patients chose to use it. During treatment, no advantage in the efficacy of a certain drug was found, and all three drugs were not found to have grade III-IV adverse reactions.

In the present study, patients with targeted therapy combined with radiotherapy after BM had the best survival advantage. The proportion of patients with CR or PR following BMs was significantly different across treatment groups. The proportion of CR + PR was 63.0% for radiotherapy, 26.7% for chemotherapy, 50.0% for targeted therapy, and 89.7% for targeted therapy combined with radiotherapy. After BM, the median survival of the four treatment groups was 20, 9, 12, and 25 months, respectively (*P* < 0.05), and their median iPFS were 12, 7, 10, and 21 months, respectively. The prognosis of chemotherapy was the worst, similar to a previous report [28]. This is thought to be due to several factors, including the blood-brain barrier (BBB) and the inherent chemotherapy resistance of BM. Thus, WBRT has been used as a standard treatment in NSCLC patients with BM, resulting in an OS ranging between 3 and 6 months since the 1970s [29, 30].

TKIs are small molecules and have a good lipid-water partition coefficient. They are easily absorbed and cross the BBB. Brain metastases can damage the BBB to some extent [31]. More recently, TKI therapy for BM patients with *EGFR* mutations achieved effective rates of 70–80% [32] and 87.8% [33]. Furthermore, the iPFS was 14.5 months, and the OS was 21.9 months. Nearly half of the patients delayed radiation therapy for more than 1.5 years after the diagnosis of BMs by using TKI [33]. Accordingly, some experts pointed out that TKI was becoming a favorable treatment, especially for patients with *EGFR* mutation of BMs of lung cancer. In the present study, the radiotherapy group did show some advantages over the targeted treatment group, probably because most patients had no more than three BMs whose maximum diameter < 2 cm, and they accepted stereotactic radiotherapy. Omuro et al. [34] and Park et al. [32] also drew similar conclusions, pointing out that TKI therapy for NSCLC brain metastases leads to a high intracranial recurrence rate and short PFS. The retrospective analysis by Magnuson et al. [35] showed that radiotherapy, compared with TKI treatment, contributed to a longer survival (34.1 vs. 19.4 months). PET/CT images with ^11^C-erlotinib as the tracer combined with the MRI images show a significant concentration of ^11^C-erlotinib in the brain metastases, but no ^11^C-erlotinib could be found in the normal brain tissues [36]. In mouse models, compared with other EGFR-TKIs, osimertinib reaches a higher concentration in the brain and is easier to accumulate in the brain [37]. In eight healthy adult volunteers (52 ± 8 years old), PET-CT was used to observe the distribution of ^11^C-osimertinib in the brain after a single intravenous injection of 1.2 μg (1.1–1.4 μg) over 90 min. It was found that ^11^C-osimertinib could distribute rapidly in the brain, with an average Tmax of 13 min and a brain/plasma AUC_0–90 min_ ratio of 8.3 ± 0.3 [38]. In the AURA3 study, the ORR of the central nervous system was 70% in the 80-mg osimertinib group and 31% in the platinum-containing dual drug chemotherapy group [39]. The ASTRIS open-label, real-world, international single-arm treatment study aimed to explore the efficacy and safety of osimertinib in T790M-positive advanced NSCLC adult patients with EGFR-TKI treatment history. The results showed that for advanced NSCLC patients with the T790M mutation and asymptomatic stable CNS metastasis treated with osimertinib, the overall ORR of T790M positive was 55% and the median PFS was 9.7 months [40]. Therefore, osimertinib could have particular benefits for patients with NSCLC and brain metastases. The role of radiotherapy in the treatment of BMs still requires additional studies, and its timing in relation to different TKIs requires additional study. Nevertheless, some studies suggested that upfront TKI and radiotherapy achieved better survival than TKI alone in patients with BMs from NSCLC [41–43].

TKIs have a radio-sensitizing effect [44, 45], and radiotherapy can disrupt the BBB to improve TKI levels in the intracranial space, and this mechanism provides a theoretical basis for the idea of targeted treatment combined with radiotherapy [46]. Zeng et al. [47], Cai et al. [48], and Welsh et al. [49] supported the hypothesis that TKI combined with WBRT is more effective for the control of intracranial lesions and prolonging the survival than either therapy alone. The benefits seemed exceptionally high for patients with *EGFR* mutation rates. Taken together, the present provides a comprehensive comparison of the various treatments. Larger prospective randomized clinical trials are needed to validate our findings and confirm these suppositions.

### Strengths and limitations

Because few of the published prognostic classification models have involved patients with *EGFR* mutation-positive NSCLC and brain metastases, the present study targeted this group of patients, trying to find out the factors affecting the prognosis and a better way treatment. In addition, the study made an overall comparison among the therapeutic effect of different treatments after BM diagnosis.

Nevertheless, there are limitations to this study. First, this study is a retrospective analysis with a relatively small number of cases and a limited follow-up duration. Second, previous studies reported that about 20–30% of patients with *EGFR* mutations are smokers [2, 3, 50, 51], compared with 37.2% in this article. This discrepancy might be related to the fact that this was a retrospective study with all the inherent biases, and that the number of cases is limited. Third, we included patients with parenchymal BMs but did not examine the exact nature of the extracranial lesions or the control of chest lesions. Fourth, the study factors are limited to general clinical factors and therapeutic factors. The exact dose and duration of treatment were not taken into account. Fifth, individualized hematology indexes and molecular indicators were not assessed. Finally, a large number of variables were included in the multivariable analysis and could make the associations inaccurate because of the small number of patients [52]. The present study should be seen as a preliminary study that tried to identify factors that could be associated with survival in a very selected population of patients, but those factors cannot be used directly to manage patients and need to be confirmed. Therefore, this study cannot provide a comprehensive reflection of the emergence, development, and prognosis of BMs in those patients. Improved data collection and/or a randomized controlled study are necessary to further examine these questions.

## Conclusions

In conclusion, the prognosis of the patients with NSCLC harboring *EGFR* mutation and BMs may be related to the number of metastatic brain lesions and the treatment methods of BMs. TKI, combined with radiotherapy, may have some advantages over other treatments in those patients. Larger prospective randomized clinical trials are needed to validate our findings and confirm these results.

## Data Availability

The datasets used and analyzed during the current study are available from the corresponding author on reasonable request.

## Abbreviations

RTOG: Radiation Therapy Oncology Group
BMs: Brain metastases
EGFR: Epidermal growth factor receptor
NSCLC: Non-small cell lung cancer
TKI: Tyrosine kinase inhibitor
OS: Overall survival
iPFS: Intracranial progression-free survival
CI: Confidence interval
RPA: Recursive partitioning analysis
WBRT: Whole-brain radiotherapy
SRS: Stereotactic radiosurgery
MRI: Magnetic resonance
CR: Complete response
PR: Partial response
SD: Stable disease
PD: Progressive disease
ORR: Objective response rate
CTCAE: Common Terminology Criteria for Adverse Events
BBB: Blood-brain barrier
